# Nanomaterials for Zinc Batteries—Aerogels

**DOI:** 10.3390/nano15030194

**Published:** 2025-01-26

**Authors:** Hulong Ruan, Zeyuan Li, Qixing Jia, Junjun Wang, Lina Chen

**Affiliations:** 1 College of Physics and Materials Science, Changji University, Changji 831100, China; 2 School of Materials Science and Engineering, Harbin Institute of Technology (Shenzhen), Shenzhen 518055, China; 3 Xinjiang Key Laboratory of High Value Green Utilization of Low-Rank Coal, Changji 831100, China

**Keywords:** nanomaterial, ZIBs, aerogel

## Abstract

Aqueous zinc batteries, mainly including Zn-ion batteries (ZIBs) and Zn–air batteries (ZABs), are promising energy storage systems, but challenges exist at their current stage. For instance, the zinc anode in aqueous electrolyte is impacted by anodic dendrites, hydrogen and oxygen precipitation, and some other harmful side reactions, which severely affect the battery’s lifespan. As for traditional cathode materials in ZIBs, low electrical conductivity, slow Zn^2+^ ion migration, and easy collapse of the crystal structure during ion embedding and migration bring challenges. Also, the slower critical oxygen reduction reaction (ORR), for example, in ZABs shows unsatisfactory results. All these issues greatly hindered the development of zinc batteries. Aerogel materials, characterized by their high specific surface area, unique open-pore structure formed by nanoporous structures, and excellent physicochemical properties, have a positive role in cathode modification, electrode protection, and catalytic reactions in zinc batteries. This manuscript provides a systematic review of aerogel materials, highlighting advancements in their preparation and application for zinc batteries, aiming to promote the future progress and development of aerogel nanomaterials and zinc batteries.

## 1. Introduction

Given that fossil fuels are highly detrimental to resources and the environment, there is an urgent need to develop a new energy reserve system that is efficient, safe, and environmentally friendly. Currently, secondary batteries, as one of the most favored energy storage systems, are widely noted [1,2]. However, achieving large-scale application in energy storage requires electrode materials that are not only economically feasible but also guarantee the long-term sustained stability and operational safety of the entire system. Zinc batteries, with their abundant resources and high safety, are regarded as one of the most promising future energy storage systems. However, during the charge/discharge cycle, dendrites—or tree-like protrusions—form on the surface of the electrodes due to uneven zinc ion accumulation at the negative electrode. The dendrites can pierce through the battery’s diaphragm, ultimately leading to a short-circuit in the battery. Collectively, these issues can significantly reduce the battery’s reversible capacity, coulombic efficiency, and cycling stability [3,4,5,6,7,8,9,10].

Aerogels constitute a category of highly porous, three-dimensional nanostructured materials that are distinguished by their high specific surface area, significant porosity, minimal refractive index, low sound velocity, low dielectric constant, negligible density, and low thermal conductivity. The principal techniques used in the preparation of aerogels include sol–gel synthesis, supercritical fluid drying, gel injection molding, and three-dimensional (3D) printing methodologies [11,12,13,14,15,16].

Nano-aerogels are solid materials characterized by three-dimensional nanoporous networks. The thermal insulation properties of ionic gels, which usually have higher ionic conductivity and some mechanical strength, may be inferior to those of aerogels. In zinc-ion batteries (ZIBs), the excellent thermal insulation performance of aerogel can more effectively prevent heat loss and battery overheating, thus enhancing the safety and stability of the battery. Moreover, the lightweight nature of aerogel can effectively reduce the weight of the battery pack, thereby enhancing its energy density. In comparison with organogels, which usually have better flexibility and processability, aerogels may not be as flexible and processable but are more thermally and chemically stable. In ZIBs, the high thermal and chemical stability of aerogels allows them to maintain stable performance at elevated temperatures and in harsh chemical environments, thus extending the battery’s service life. Also, the nanoporous structure of the aerogel provides excellent adsorption properties and ion transport channels, which helps improve the charging and discharging performance and energy density of the battery.

Based on their chemical composition, nano-aerogels can be classified into two types: inorganic and organic. Inorganic nano-aerogels are mainly used for reinforcement purposes and are usually combined with inorganic fiber cloths or polymeric materials. In contrast, organic nano-aerogels possess excellent mechanical properties such as high hardness, high strength, high toughness, and considerable flexibility. This is because their molecular structures can be precisely tailored at the nanoscale, allowing for the manipulation of intermolecular forces and material properties. The excellent properties of nano-aerogels have led to their extensive application in a variety of energy storage batteries [17,18,19,20,21,22,23,24,25].

## 2. Aerogel Preparation Methods

Aerogels constitute a distinctive category of nanomaterials. Their exceptionally high porosity and remarkably low density have attracted considerable notice [26,27]. Essentially, they are solid materials composed of a gas dispersed within a gel network structure, representing one of the least dense solid materials known to date [28]. Aerogels possess the following characteristics: Ultra-low density: The density of aerogels can be as low as 0.16 mg cm^−3^, which is approximately one-sixth of the density of air [29,30], making them among the lightest solid materials known. Low thermal conductivity: Aerogels exhibit extremely low thermal conductivity, with values as low as 10^−3^ W·m^−1^·K^−1^ at environmental temperature under vacuum conditions [31,32]. This property makes them highly effective thermal insulators. High porosity: Aerogels are characterized by a highly porous internal structure, with pores predominantly in the nanometer range. As can be seen, these pores pervade the entire volume of the material [33,34], resulting in an extraordinarily high surface area-to-volume ratio. Porous structure: The interconnected porous network of aerogels significantly impedes heat transfer, contributing to their excellent thermal insulation properties [35,36]. This unique structure allows for the trapping of air within the pores, further enhancing their insulating capabilities [37,38].

**Figure 1:**
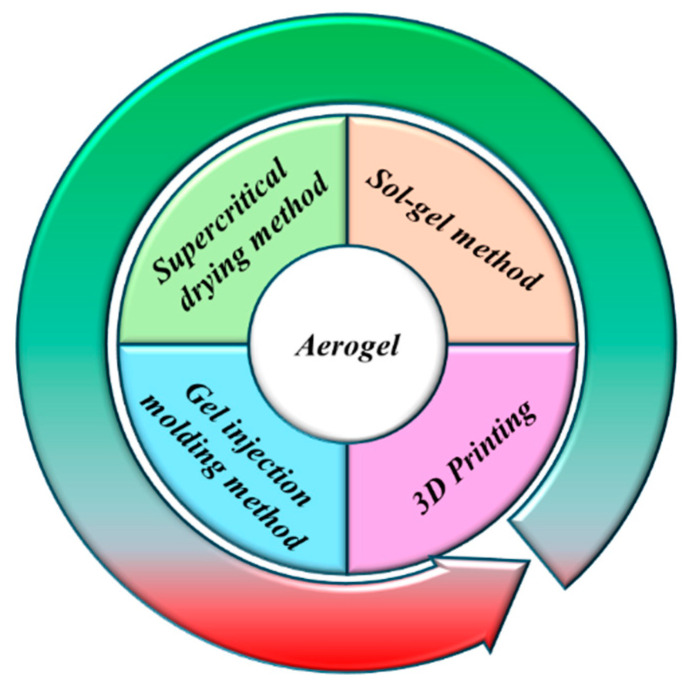
Four common methods for preparing aerogels.

In AZBIs, the growth of Zn dendrites has been one of the most significant challenges encountered to date [39,40]. Aerogels, however, can address this issue by homogenizing the flux of Zn^2+^ ions during the electrochemical process [41,42,43], thereby enabling rapid and uniform zinc deposition. Simultaneously, they effectively regulate the interfacial zinc ion desorption behavior, thereby bolstering the Zn anode’s stability. The enthusiasm of researchers towards aerogels is also attributed to their ability to improve interfacial stability, enhance electrolyte performance, and suppress side reactions. Figure 1 illustrates the four common preparation processes for aerogels. The main methods for preparing aerogels include the sol-gel method [44,45], supercritical drying method [46,47], gel injection molding method [48,49], and three-dimensional printing [50,51]. The following sections provide a detailed description of these methods. The four preparation methods each have their own advantages and disadvantages. Table 1 summarizes the preparation process description, advantages, and current limitations of each method. Different preparation processes have distinct applications in zinc batteries. Table 2 summarizes the applications of the four processes in this field.

### 2.1. Sol–Gel Method

**Figure 2:**
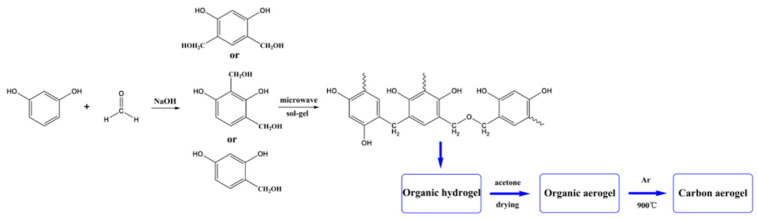
The process of preparing the aerogel KOH-CA using the microwave-assisted sol–gel method [52]. Reproduced with permission. Copyright 2019, MDPI.

As a wet chemical synthesis technique, the sol–gel method mainly consists of the hydrolysis and polycondensation reactions of metal alkoxides or inorganic compounds within a liquid environment to generate a sol. This sol subsequently undergoes aging and drying processes to transform into a gel, ultimately resulting in the formation of an aerogel product. Cai et al. [52] resorted to a microwave-facilitated sol–gel method for producing hierarchical porous carbon aerogels. Figure 2 outlines the preparation methodology as follows: with resorcinol and formaldehyde serving as the raw materials and sodium hydroxide acting as the catalyst, an RF organic aerogel was produced through the sol–gel method at the temperature of 85 °C and a power of 100 W. Subsequently, the gel’s surplus solvent was substituted with acetone, after which the drying process was carried out. The organic aerogel was pyrolyzed in a nitrogen atmosphere for 2 h to carbonize it, resulting in the formation of CA. Finally, CA was mixed with KOH and activated at 800 °C for 2 h in a nitrogen atmosphere. After washing and drying, the activated carbon aerogel (KOH-CA) with a substantial specific surface area and excellent electrochemical performance was obtained [53,54].

### 2.2. Supercritical Drying Method

**Figure 3:**
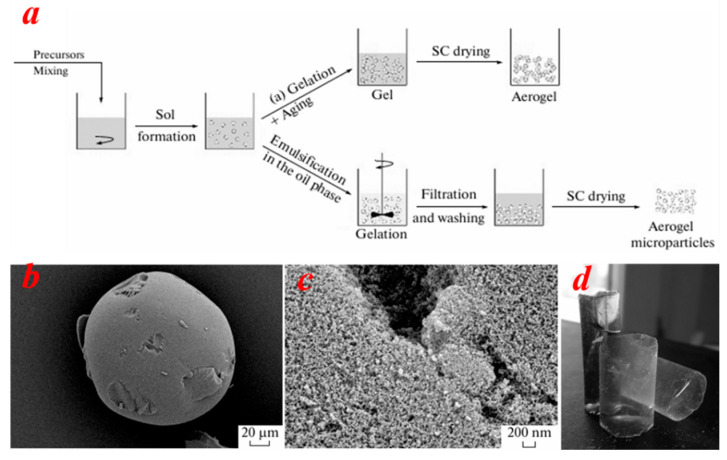
(**a**) Chemical preparation process schematic of the aerogel SC-CO_2_ [57]. (**b**,**c**) SEM images of SC-CO_2_ [57]. (**d**) Image of a cylindrical aerogel sample [57]. Reproduced with permission. Copyright 2024, Springer Nature.

Supercritical drying offers a number of advantages, ensuring structural integrity, significant specific surface area and porosity, and relatively short drying times [55,56]. Consequently, it finds extensive application in various domains including thermal insulation, catalysis, and energy storage systems. N. V. Men’shutina et al. [57] employed the supercritical drying method to prepare silica aerogels. The process involved dissolving tetraethoxysilane (TEOS) in ethanol and adding citric acid as a catalyst to form a sol. Subsequently, ammonia water was introduced to initiate the condensation reaction, leading to gel formation. After gelation in a mold, the alcogel was soaked in anhydrous ethanol to remove unreacted precursors. The alcogel was then placed in a specially designed high-pressure reactor, where it was subjected to supercritical carbon dioxide (SC-CO_2_) at a pressure of 120–150 atm and at 40 °C. The reactor’s design was optimized to ensure uniform distribution of SC-CO_2_, ultimately completing the drying process. The resulting silica aerogel exhibited a high specific surface area of 926.7 m^2^/g, a low density of 0.04 g cm^−3^, and a typical porous network structure. SEM images of the sample are presented in Figure 3b,c. The synthesized aerogel has a porous morphology, and under high resolution, the aerogel presents a regular spherical shape. Figure 3d is a display diagram of a cylindrical aerogel sample.

### 2.3. Gel Injection Moulding Method

**Figure 4:**
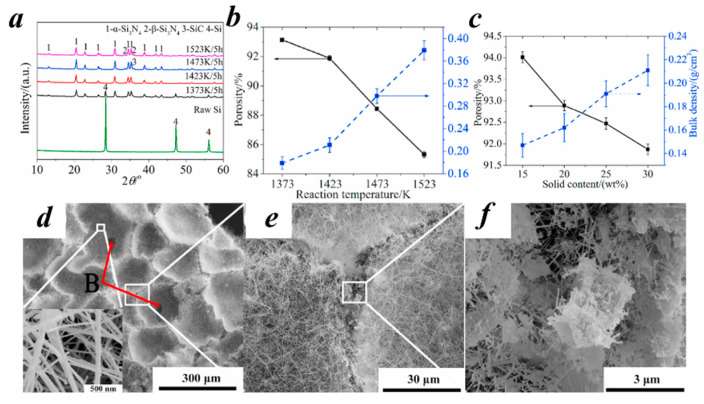
(**a**) The XRD pattern corresponding to the SCSNCAs aerogel. (**b**) The effect of the SCSNCAs reaction temperature on porosity. (**c**) The effect of the solid composition on the SCSNCAs aerogel. (**d**–**f**) SEM images of the SCSNCAs aerogel at different magnifications [48]. Reproduced with permission. Copyright 2024, Elsevier.

Gel injection molding is a fabrication method that involves injecting a gel into a mold to produce a gel product with specific morphological requirements or special structures, with the core principle being the utilization of the gel’s fluidity [52,58,59]. The process includes the following steps: Sol preparation: The sol is typically composed of three components: metal alkoxides or metal-organic compounds, a solvent, and a surfactant. Mold injection: The sol is injected into a mold, which can be customized to achieve the desired shapes and dimensions. Gelation: The sol within the mold undergoes a transformation into a gel through chemical reactions or physical changes. This process can be accelerated using heating, chemical reactions, or photopolymerization. The drying process for gels can be carried out using various techniques, such as drying at atmospheric pressure, in a supercritical state, or by freezing technology. Gel injection molding offers several advantages, including the ability to produce diverse shapes, a simple process, and excellent material properties. As a result, it is widely used in the production of thermal insulation materials and composite materials. Han et al. [48] used a rotary mill to mix silicon powder and melamine, and then dispersed the mixture in 30 wt% deionized water for uniform mixing. The resulting slurry was transferred into the appropriate mold and subsequently dried under high-temperature conditions. Heating in a N_2_ atmosphere to 1523 K and holding for 5 h yielded the aerogel product SCSNCAs. Its XRD characterization is shown in Figure 4a. The temperature and load affect the porosity and structural characteristics of the synthesized material. As shown in Figure 4b,c, the optimal combination was obtained at a temperature of 1423 K and a load of 30 wt%, yielding an aerogel material with a porosity of 91.9%. This porosity is evidenced by the SEM images shown in Figure 4d–f.

### 2.4. Three-Dimensional Printing

**Figure 5:**
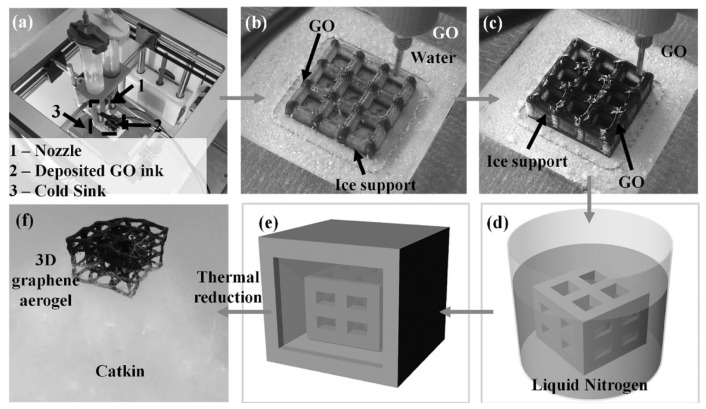
Schematic diagram of the preparation process for 3D printed CA aerogel. (**a**) Initialization of printing parameters: Setting up the initial values for the 3D printing process. (**b**) Ice scaffold-assisted printing of CA aerogel: Using an ice scaffold as a support structure during the 3D printing of the CA aerogel. (**c**) Schematic of graphene oxide (GO) printing: Illustrating the process of printing graphene oxide (GO) layers. (**d**) Schematic of liquid nitrogen curing process: Showing the step where liquid nitrogen is used to solidify the printed structure. (**e**) Schematic of freeze-drying process for CA aerogel: Depicting the freeze-drying process to remove moisture from the CA aerogel. (**f**) Schematic of thermal reduction for CA aerogel: Illustrating the thermal reduction process to enhance the properties of the CA aerogel [63]. Reproduced with permission. Copyright 2024, John Wiley and Sons.

Three-dimensional molding technology, also known as additive manufacturing or what we know as 3D printing, is an advanced technique that fabricates three-dimensional objects by successively layering material. In recent years, 3D printing has made significant progress in the preparation of aerogels. This technology enables the fabrication of aerogels with complex geometric shapes, which are hard to achieve through conventional production processes [60,61,62]. Additionally, 3D printing allows for the customization of aerogel designs, enabling the adjustment of aerogel structures and properties according to experimental requirements. Three-dimensional printing can significantly reduce the preparation time of aerogels and enhance the overall efficiency of the fabrication process. The preparation process primarily includes ink formulation, the printing process, drying, and post-processing. The composition and rheological properties of the ink have a substantial impact on the printing outcome. Zhang et al. [63] resorted to 3D printing for producing graphene aerogels (GAs) with specific macrostructures and high performance. The preparation process is shown in Figure 5. Single-layer graphene oxide was used as the precursor, and GO powder was dispersed in deionized water to prepare a uniform suspension. The material was prepared using low-temperature 3D printing technology. Post-printing, the material was dipped into liquid nitrogen to undergo freeze-drying, and finally, thermal reduction was performed at 1000 °C for 30 min to obtain the final graphene aerogel (GA) product.

**Table 2:** Applications in zinc batteries of four methods for preparing aerogels.

Preparation Method	Potential Applications in Zinc Batteries
Sol–Gel Method	1. Ideal for synthesizing electrode materials with high porosity and surface area. 2. Suitable for developing zinc–air battery catalysts.
Supercritical Drying Method	1. Suitable for preparing high-performance electrode materials that require precise control over porosity and structure. 2. Can be used in zinc-ion capacitors for improved ion transport.
Gel Casting Method	1. Useful for fabricating customized electrode structures for zinc batteries. 2. Can be applied in the production of zinc-based flow batteries with specific geometries.
3D Printing	1. Enables the fabrication of intricate electrode architectures for zinc batteries. 2. Facilitates the development of high-performance zinc-ion batteries with tailored porosity and surface area.

## 3. Application of Aerogel in Zinc-Ion Batteries

Recently, the use of aerogel nanomaterials in a variety of energy conversion and storage systems has been increasing substantially, and these include dye-sensitized photovoltaic cells, water decomposition equipment, power storage systems, power combustion units, and high-performance capacitors. In the construction of these energy conversion and storage devices, the adsorption performance and ion transport efficiency are regarded as the key factors determining the high performance of electrochemical energy devices. Therefore, aerogel nanomaterials have become a key material in the field of energy storage due to their good properties [64,65,66,67,68]. Similarly, they are widely used in zinc-ion batteries.

### 3.1. Anode Material Modification

Zinc-ion batteries are one of the most popular energy storage systems at present. However, during their charge/discharge cycles, the negative electrode undergoes a continuous process of accumulating two-dimensional zinc ions, which can form tree-like protrusions known as dendrites on the electrode surface. These dendrites have the potential to penetrate the separator material of the battery, resulting in an internal short-circuit. Furthermore, once the dendrites are dislodged from the negative electrode and enter the electrolyte, they become electrochemically inactive, referred to as ‘dead zinc’. Simultaneously, the immersion of the zinc electrode in the electrolyte triggers a vigorous hydrogen precipitation reaction. In essence, these issues significantly impact the battery’s cycling capacity, coulombic efficiency, and overall stability [69,70].

**Figure 6:**
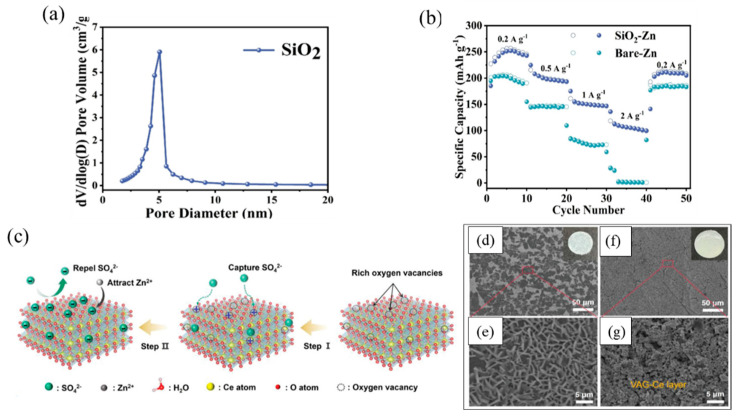
(**a**) Pore size distribution of SiO_2_ aerogel powder. (**b**) Rate performances of bareZn//MnO_2_ and SiO_2_−Zn//MnO_2_ full batteries at varies current density [71]. Reproduced with permission. Copyright 2024, John Wiley and Sons. (**c**) Protective effect of the VAG-Ce layer and role description images. SEM images of (**d**,**e**) bare Zn anode and (**f**,**g**) VAG-Ce@Zn anode after 24 h of cycling [41]. Reproduced with permission. Copyright 2024, John Wiley and Sons.

Zhang et al. [71] employed sol–gel technology to create an aerogel material containing a variety of polar functional groups. Figure 6a shows that the silica aerogel possesses a specific surface area of up to 725 m^2^/g. The abundant porous structure significantly enhances the penetration efficiency of the electrolyte, which in turn hinders the formation of 2D dendritic structures during the cycling process and achieves the enhancement of electrochemical performance. In addition, the abundant hydroxyl groups on the SiO_2_ surface not only effectively bind Zn^2+^ in the electrolyte and reduce the overpotential of zinc nucleation, but also promote the migration of hydrated zinc ions and accelerate the kinetics of zinc deposition. Therefore, the layered structure of the SiO_2_ aerogel guides the efficient deposition of zinc and is inhibitory to the formation of dendritic dendrites and other harmful invalid reactions in the zinc anode. As demonstrated in Figure 6b, when testing the performance of anode-assembled cells at different rates ranging from 0.2 A/g to 2 A/g, the SiO_2_-Zn anode exhibits superior rate capability over the bare Zn anode at all tested current densities. Particularly noteworthy is that the SiO_2_-Zn//MnO_2_ cells were able to maintain good cycling stability and high-capacity performance at current densities as high as 2 A/g. Compared to bare zinc/manganese dioxide batteries, the capacity degradation of bare zinc/manganese dioxide batteries is obvious. In addition, side effects during circulation are also an issue that needs to be given sufficient attention [72,73]. The by-product Zn_4_SO_4_(OH)_6_-xH_2_O(ZSH) is produced by the side reaction, which can severely damage the flatness of the electrode surface, further promote the growth of dendritic structures, and adversely affect the migration of Zn^2+^ ions [74].

Liu and his colleagues [41] pioneered a CeO_2_ nano-aerogel containing abundant oxygen vacancies (referred to as ag-ce) as an interfacial layer to stabilize the zinc anode in rechargeable zinc-ion batteries (ZIBs). The ag-ce protective layer possesses a well-defined and homogeneous nano-channel structure, which not only promotes the fast and homogeneous transport of Zn^2+^ ions but also is effective in regulating the Zn^2+^ ion flux on the zinc negative electrode. In addition, the oxygen-deficient sites enriched on the ag-ce show a strong adsorption of sulfate ions (SO_4_^2−^); this property makes the negatively charged coating produce an electrostatic effect, which effectively attracts Zn^2+^ ions around the zinc anode while repelling SO_4_^2−^ ions, thus eliminating the side reaction from the source and accelerating the migration process of Zn. Excitingly, ag-ce has a high surface area of 141.953 m^2^/g, which ensures a large amount of exposure of active sites as well as a sufficient contact area with the electrolyte. As shown in Figure 6c, some defective structures consisting of oxygen-deficient sites marked by red circles can be observed in ag-ce. The more oxygen vacancies there are, the more the effective sites for capturing SO_4_^2−^ increase, which reduces the concentration of SO_4_^2−^ near the surface of the Zn anode and effectively inhibits the ZSH generation of by-products, thereby reducing the side reactions. Ultimately, we conducted a comparative analysis of the surface appearance of zinc anodes following a 24 h cycling period at a current density of 1 mA/cm^2^. As shown in Figure 6d,e, a large number of dendritic agglomerates and by-products existed on the surface of the bare zinc anode. Digital photographs of the bare zinc anode after cycling visually show the severe corrosion it suffered. In contrast, as shown in Figure 6f,g, the surface morphology of VAG-Ce@Zn before and after cycling remained highly consistent, with no obvious dendrite or by-product formation observed. This sharp contrast fully demonstrates the outstanding performance of VAG-Ce@Zn in promoting the uniform deposition of zinc and inhibiting the side reactions. After cycling tests on the cells, the results show that VAG-Ce@Zn can still maintain excellent discharge capacity of 130 mAh/g after 1000 cycles, and the average coulombic efficiency is over 99.5%.

### 3.2. High Performance Cathodes

Various substances are currently available as cathode materials for zinc-ion batteries, including manganese dioxide (MnO_2_), metal-organic framework materials (MOFs), two-dimensional organic frameworks (COFs), quinone compounds, polyester fibers, polyaniline, graphene-based materials, and others. However, all these materials encounter several problems and challenges. For instance, MnO_2_, when used as a cathode material in zinc-ion batteries (AZIBs), has long faced the problem of significant degradation of specific capacity at high load qualities, which hinders its development and application. Additionally, COFs exhibit poor stability and cycling performance. Furthermore, the application of quinone compounds, polyester fibers, polyaniline, and other materials as cathode materials in zinc-ion batteries is still in the research stage and requires further optimization [74,75,76,77,78].

**Figure 7:**
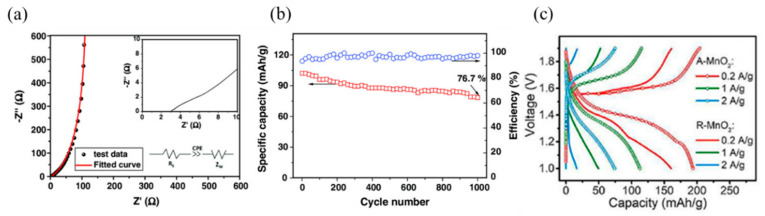
(**a**) EIS curve diagram. (**b**) Cycling performance image of 1000 charge/discharge cycles at a current density of 8 A g^−1^ [79]. Reproduced with permission. Copyright 2024, John Wiley and Sons. (**c**) GCD images at various current densities [84]. Reproduced with permission. Copyright 2024, Elsevier.

Li et al. [79] has developed a composite aerogel based on nanostructured polypyrrole (PPy), which can be used as a flexible cathode for zinc-ion batteries. The composite aerogel contains polyvinyl alcohol (PVA), which has good physical properties and provides a solid matrix for the in situ polymerization of PPy. The resulting nanostructured PPy composite aerogel exhibits good flexibility and can be easily bent, making it ideal for use as a flexible cathode material. In Figure 7a, the loose porous structure of the composite aerogel can be observed, and ions can effectively penetrate into the nanostructured PPy to improve electrical conductivity. The electrochemical test results showed that the energy density of the cell was as high as 64.0 Wh/kg and the power density was superior compared to other water batteries and supercapacitors. In addition, as shown in Figure 7b, it was found that by observing the battery capacity data after 1000 charge/discharge cycles, the battery capacity retention rate was still as high as 76.7% and the coulombic efficiency remained above 95%.

For decades, manganese oxides, especially manganese dioxide, have been widely used in zinc-ion batteries (ZIB) due to the abundance of natural resources available, non-toxicity, flexible redox reactivity, high redox potentials, remarkable theoretical capacity (specifically, manganese dioxide’s theoretical capacity reaches 308 mAh/g), easy synthesis, and tunable crystal structure, among other advantages. It is considered the most promising cathode material for zinc-ion battery (ZIB) systems [80,81]. However, during the embedding and de-embedding of Zn^2+^, manganese dioxide cathodes have inherently high resistivity, slow Zn^2+^ diffusion kinetics, and an easily collapsible crystal structure [82,83]. Dong and his colleagues report a scalable synthetic method for the preparation of nano-aerogel structures of MnO_2_ (denoted as A-MnO_2_) [84] and the A-MnO_2_ material was constructed from defect-rich ultrathin nanosheets, which were prepared by a V_2_O_5_ gel-assisted facile co-precipitation method. The electronic structure of A-MnO_2_ was optimized by doping vanadium (V) elements and inducing the generation of oxygen vacancies, which not only reduces its resistivity but also improves the diffusion kinetics of Zn^2+^ ions. In addition, the aerogel structure consisting of ultrathin nanosheets was able to expose more electrochemically active sites and shorten the diffusion path of ions. As a result, the electrochemical kinetic performance of A-MnO_2_ was significantly improved. In the application of cathode materials for zinc-ion batteries (ZIBs), A-MnO_2_ has been optimized for several performance metrics such as battery capacity, charge/discharge rate, and cycle durability compared to defect-free manganese dioxide nanorods. It can be found by observing the electrostatic charge/discharge (GCD) test results presented in Figure 7c that the discharge capacity of the cathode is 161 mAh/g at a current density of 0.2 A/g; the discharge capacity reaches 50 mAh/g at 1 A/g and 17 mAh/g at 2 A/g. In addition, when the current density was restored to 0.2 A/g, the discharge capacity of the A-MnO_2_ cathode was increased to 194 mAh/g, and the capacity of 74 mAh/g was maintained even at the high current density of 2 A/g. This performance is significantly better than that in other related reports of MnO_2_ materials in pure ZnSO_4_ and unmodified electrolytes.

### 3.3. Highly Efficient Electrocatalysts

**Figure 8:**
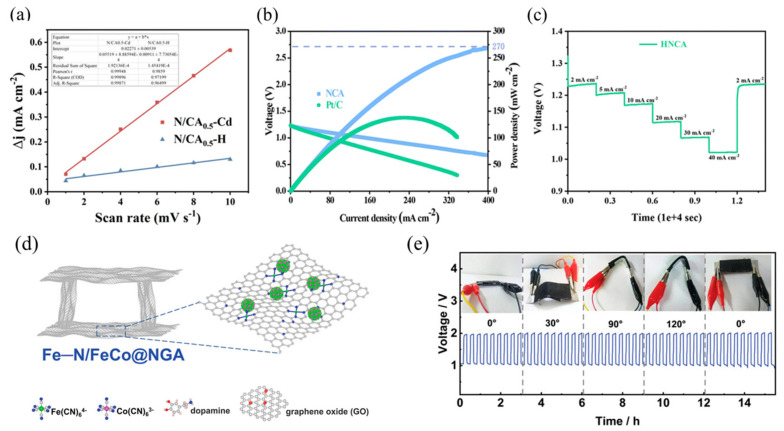
(**a**) Image of current density difference (Δj) versus scan rate [88]. Reproduced with permission. Copyright 2022, American Chemical Society. (**b**) Discharge polarization curves and corresponding power density images of ZAB with Pt/C+RuO_2_ and HNCA as air electrodes. (**c**) Images of discharge curves of HNCA-based ZAB at different current densities [90]. Reproduced with permission. Copyright 2024, Portion. (**d**) Schematic diagram of Fe—N/FeCo@NGA defects. (**e**) Charge/discharge cycles of Fe-N/FeCo@NGA ZAB at different bending angles [91]. Reproduced with permission. Copyright 2025, Small.

In the realm of energy transformation methods utilizing electrochemical principles, especially those involving fuel cells and metal–air batteries, the oxygen reduction reaction (ORR) holds a critical yet typically slow position as a vital component in the overall process. However, the widespread use of precious metal electrocatalysts in commercial applications is limited by the fact that they are not only expensive, but also suffer from significant performance degradation after long-term operation [55,56,57,58,59,60,61,62,63,64,65,66,67,68,69,70,71,72,73,74,75,76,77,78,79,80,81,82,83,84,85,86,87]. Liu and colleagues [88] designed an innovative method for the synthesis of TOCNF/ANF-Cd nano-aerogels, thermally resilient aramid nanofibers (ANFs), and Cd^2+^ with a low boiling point. Then, they synthesized TOCNF/ANF synergistically crosslinked N/CA-Cd multistage porous aerogel catalysts by applying a medium-temperature pyrolysis technique. During the pyrolysis process, TOCNF and ANF underwent volatilization, resulting in the formation of crosslinked carbon nanofibers with a distinctive porous structure. This structure facilitates the rapid diffusion of ions in the reaction while enhancing proton conductivity. Additionally, nitrogen atoms derived from ANF were partially incorporated within the carbon lattice structure, while surface irregularities and cavity defects were introduced into the carbon framework. These defects disrupted the coherence of the π-conjugation system, modifying the electronic properties of the resultant material [89]. During the oxygen reduction reaction (ORR), carbon atoms exhibit charge polarization and are able to strongly adsorb oxygen-containing substances, which in turn accelerates the reaction process. As shown in Figure 8a, the Tafel slopes of 71 mV dec^−1^ and 77 mV dec^−1^ exhibited by N/CA0.5-Cd compared to the standard 20 wt% Pt/C catalyst are strong evidence of the superior performance of N/CA0.5-Cd in ORR kinetics. Zn–air cells equipped with N/CA0.5-Cd air cathodes have a maximum power density of up to 186 mW cm^−2^, which significantly outperforms that of the commercially available Pt/C-RuO_2_ catalyst (135 mW cm^−2^).

In addition, Wei et al. successfully prepared nitrogen-doped porous carbon nano-aerogels by combining hydrothermal pretreatment with a carbonization process [90]. During the synthesis process, carbon nanofibers (CNFs) served as the structural backbone of the aerogel, facilitating the construction of an interconnected porous network. Meanwhile, graphitic carbon nitride (g-C3N4) can be used as an in situ nitrogen doping source while promoting the generation of additional nanopores within this structure. Subsequently, the CNF/g-C3N4 aerogel synthesized by the hydrothermal method was then pyrolyzed in nitrogen atmosphere to finally obtain nitrogen-doped carbon aerogel (HNCA for short). To fabricate the zinc–air battery (ZAB), the researchers utilized a mixture comprising 6 M potassium hydroxide (KOH) and 0.2 M zinc acetate as the electrolyte. The anode is a 0.3 mm thick zinc plate and the cathode consists of HNCA or a powdered mixture of Pt/C and RuO_2_. It is coated on a carbon cloth. The ZAB employing HNCA as the cathode catalyst exhibited a peak power density of 270 mW cm^−2^, outperforming the ZAB with Pt/C + RuO_2_, as demonstrated in Figure 8b,c. During the enhancement of current density from 2 mA cm^−2^ to 40.0 mA cm^−2^, the HNCA-based ZAB exhibited a maximum power density of 270 mW cm^−2^, outperforming the ZAB that utilized Pt/C + RuO_2_ as the cathode catalyst. The HNCA cathode maintained a discharge voltage of 1.0 V even at a current density of 40.0 mA cm^−2^. When the current density was subsequently adjusted to 2 mAcm^−2^, the discharge voltage was able to be reversibly ramped back up to its initial 1.23 V level, thereby confirming the excellent performance of the HNCA. Under high-load conditions, the performance of the battery is further demonstrated by its capacity of 790.0 mAh g^−1^ at a current density of 5 mA cm^−2^, as well as its high energy density of 945.6 Wh kg^−1^.

Tang and colleagues [91] recently reported the synthesis of an N-doped graphene aerogel, specifically Fe-N/FeCo@NGA, featuring monatomic Fe-N4 and FeCo_NA_ double sites, as a high-performance ORR electrocatalyst. This material possesses moderate backbone defects within the NGA framework. Defects in the skeleton exhibit specific effects on both electronic conductivity and structural robustness. Firstly, an insufficient number of defects hinders the formation of sufficient active sites. Conversely, an excessive number of defects can adversely impact electronic conductivity and structural stability [92,93,94]. As illustrated in Figure 8d, the Fe-N/FeCo@NGA aerogel can be pyrolyzed by mixing H_2_ and Ar in equal proportions to obtain a carbon skeleton with moderate defects, effectively achieving a favorable balance between electronic conductivity and the presence of active double sites. To assess the electrochemical performance of this material, an all-solid-state zinc–air battery (ZAB) was assembled. This ZAB exhibits a superior specific capacity of 814.4 mAh g^−1^ at a discharge voltage plateau of 1.29 V. Moreover, it maintains its bending state at various angles for a duration of 3 h and undergoes continuous charge/discharge cycles at a high current density of 10 mA cm^−2^, with its round-trip efficiency remaining almost unchanged, as depicted in Figure 8e. This outcome further underscores the significant potential of Fe-N/FeCo@NGA nano-aerogel materials for application as energy storage devices.

Overall, nano-aerogels demonstrate unique effects in several scenarios of zinc batteries. Their nanostructure, high specific surface area, abundant polar functional groups, and high electrical conductivity significantly enhance electrolyte penetration, induce ion deposition, and strengthen the binding between the electrolyte and active substances. These properties address some of the current problems in zinc batteries. Nano-aerogels with abundant polar functional groups can not only effectively combine Zn^2+^ in the electrolyte and reduce the overpotential of zinc nucleation, but also promote the migration of hydrated zinc ions, accelerate the kinetic process of zinc deposition, guide the effective deposition of zinc, and inhibit the formation of dendritic dendrites and other harmful ineffective reactions in the zinc anode. In addition, the low permeability of the active substance to the electrolyte can lead to insufficient reaction between the electrolyte and the active substance, resulting in a decrease in the capacity of the battery. These are the main research directions for using aerogel solutions to address some of the current challenges of zinc batteries. However, the porous structure of the nano-aerogel that can sufficiently permeate with the electrolyte is able to solve this problem. However, the stacking density of aerogel is extremely low (usually <0.5 g/cm^3^), which leads to unsatisfactory volumetric energy density. Volumetric energy density is an important parameter in battery design, and a low density will make the battery larger, affecting practical applications. In addition, aerogels are generally fragile and easily ruptured under external pressure or impact. This fragility not only affects their long-term use in batteries, but also makes them more susceptible to damage during processing and handling. Also, there are stability issues with the electrolyte, as degradation or dissolution of the material could affect battery life [95,96,97,98].

## 4. Summary and Outlook

With its unique network structure and hydrophilic properties, aerogel shows significant potential in the field of zinc batteries. Aerogels can rapidly gelatinize the aqueous electrolyte and form a hydrogel-poor interfacial layer, which can effectively stabilize the zinc metal anode and inhibit the growth of zinc dendrites. Meanwhile, the nanoporous structure of the aerogel provides abundant active sites, which facilitates the embedding and de-embedding of Zn^2+^, enhances the zinc storage capacity, and accelerates the kinetics of the anode reaction. In addition, aerogel as a catalyst also shows unique advantages, which can significantly accelerate the key steps in the battery reaction. For example, in the oxygen reduction reaction of zinc–air batteries, aerogel-based catalysts can effectively reduce the activation energy of the reaction and increase the reaction rate, which in turn improves the overall performance of the battery. In terms of preparation, different preparation methods, such as the sol–gel method, supercritical drying, gel injection molding, and 3D printing, have their own advantages and disadvantages, providing options to meet different application requirements. Moreover, aerogels have achieved good results in the modification of anode materials, high-performance cathodes, and high-efficiency electrocatalysts for zinc-ion batteries, effectively solving some of the problems in the batteries, such as improving their cycling stability, capacity, and energy density. However, aerogels still have some shortcomings at present, such as low stacking density, leading to suboptimal volumetric energy density, fragile materials, and possible stability problems in electrolytes, which limit their wide application in batteries.

**Figure 9:**
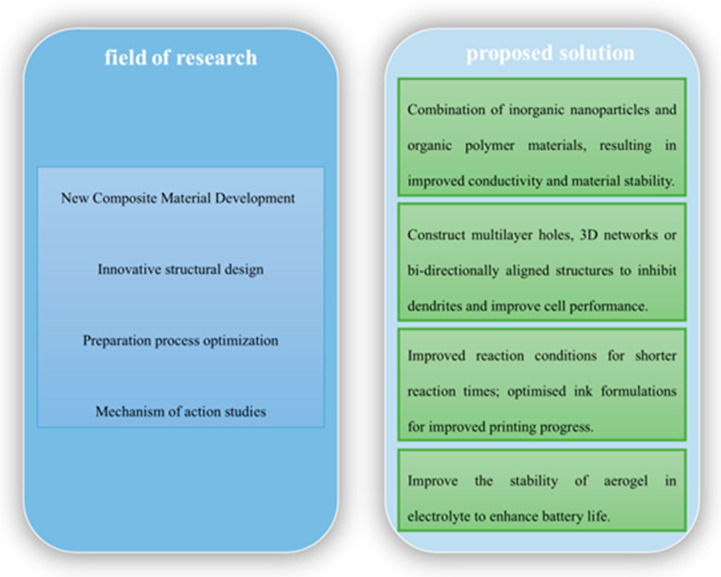
Key future research directions and challenges.

In order to promote further development in the field of zinc batteries, future research can focus on several directions. These are suggestedare shown in Figure 9: In the material research and development, actively explore and develop new aerogel composites, and rationally combine inorganic nanoparticles, organic polymers, etc., to improve the conductivity, ionic conductivity, and stability of the material. At the same time, innovate the structural design of aerogel, such as the construction of a multilayer pore structure, three-dimensional network structure, or orientation arrangement structure, which not only can increase the specific surface area and increase the ion transport channel, but also help to inhibit the formation of zinc dendrites, thus improving the comprehensive performance of the battery. In terms of the preparation process, continue to optimize the existing preparation methods, reduce production costs, improve production efficiency, and make them more suitable for large-scale industrial production. For example, improve the reaction conditions of the sol–gel method to shorten the reaction time and reduce the cost, and optimize the formulation of ink in 3D printing technology to improve the printing accuracy. In addition, conduct in-depth study of the interaction mechanism between the aerogel and electrolytes to solve the problem of the stability of the aerogel in the electrolyte and ensure the long-term stable operation of the battery. The synergistic promotion of these research directions is expected to realize the efficient, stable, and reliable application of aerogel in zinc batteries and promote the major breakthrough of zinc battery technology.

## Figures and Tables

**Table 1:** Summary of the advantages, disadvantages, and preparation processes of four aerogel preparation methods.

Preparation Method	Characteristics	Advantages	Limitations
Sol–gel method	Through hydrolysis and polycondensation reactions, a gel is formed, which is then dried to obtain an aerogel.	Uniform at the molecular level. Easy to dope. Low-temperature synthesis.	High cost. Time-consuming. Prone to shrinkage and cracking.
Supercritical drying method	Utilizes the properties of supercritical fluids to dry the gel, avoiding structural damage.	Maintains high porosity. Complete structure. Shorter drying time.	High equipment cost. Complex operation.
Gel casting method	The gel is injected into a mold, and after curing and drying, an aerogel with a specific shape is obtained.	Can prepare complex shapes. Simple process. Suitable for large-scale production.	High requirements for mold design. Requires precise control of the process.
3D printing	Manufacturing three-dimensional aerogel structures by printing materials layer by layer.	Customizable complex structures. Efficient preparation. Suitable for various applications.	Limited types of ink. Complex design of hierarchical pore structures. Printing precision needs improvement.
